# The effect of rapamycin, NVP-BEZ235, aspirin, and metformin on PI3K/AKT/mTOR signaling pathway of *PIK3CA*-related overgrowth spectrum (PROS)

**DOI:** 10.18632/oncotarget.17566

**Published:** 2017-05-02

**Authors:** Yasuyo Suzuki, Yasushi Enokido, Kenichiro Yamada, Mie Inaba, Kumiko Kuwata, Naoki Hanada, Tsuyoshi Morishita, Seiji Mizuno, Nobuaki Wakamatsu

**Affiliations:** ^1^ Department of Genetics, Institute for Developmental Research, Aichi Human Service Center, Kasugai, Japan; ^2^ Department of Pathology, Institute for Developmental Research, Aichi Human Service Center, Kasugai, Japan; ^3^ Department of Pediatrics, Central Hospital, Aichi Human Service Center, Kasugai, Japan; ^4^ Department of Plastic and Reconstructive Surgery, Aichi Children's Health and Medical Center, Obu, Japan; ^5^ Hanada Kodomo Clinic, Okazaki, Japan

**Keywords:** PIK3CA-related overgrowth spectrum (PROS), heterozygous mosaic mutation, PI3-kinase, mTOR, metformin

## Abstract

The phosphatidylinositol 3-kinase (PI3K)/AKT/mTOR signaling pathway is critical for cellular growth and metabolism. Recently, mosaic or segmental overgrowth, a clinical condition caused by heterozygous somatic activating mutations in *PIK3CA*, was established as *PIK3CA*-related overgrowth spectrum (PROS). In this study, we report a Japanese female diagnosed with PROS, who presented with hyperplasia of the lower extremities, macrodactyly, multiple lipomatosis, and sparse hair. Sequencing and mutant allele frequency analysis of *PIK3CA* from affected tissues revealed that the patient had a heterozygous mosaic mutation (c.3140A>G [p.H1047R]) in *PIK3CA* and that there were higher mutant allele frequencies from samples with a larger amount of subcutaneous adipose tissue. We established two fibroblast cell lines from the patient, harboring high and low frequencies of the mosaic mutation, in which AKT and S6 showed higher level of phosphorylation compared with three control fibroblasts, indicating that PI3K/AKT/mTOR signaling is activated. We assessed the therapeutic effects of four compounds (rapamycin, NVP-BEZ235, aspirin, and metformin) on PI3K/AKT/mTOR signaling pathway and cell growth. All four compounds suppressed S6 phosphorylation and inhibited cell growth of the patient-derived fibroblast cell lines. However, only metformin mildly inhibited the growth of the control fibroblast cell lines. Since PROS is a congenital disorder, drugs for therapy should take into consideration the natural growth of children. Thus, metformin is a candidate drug for treating PROS in growing children.

## INTRODUCTION

The somatic activating mutations in *PIK3CA* encoding the phosphatidylinositol-4,5-bisphosphate 3-kinase catalytic subunit alpha (PIK3CA, also known as p100α) cause heterogeneous mosaic or segmental overgrowth disorders including fibroadipose overgrowth (FAO) [1], hemihyperplasia multiple lipomatosis (HHML) [2], congenital lipomatous overgrowth, vascular malformations, epidermal nevi, scoliosis/skeletal and spinal (CLOVES) syndrome [3–5], macrodactyly and muscle hemihypertrophy [6], megalencephaly syndromes such as megalencephaly-capillary malformation (MCAP) [7] and hemimegalencephaly [8], skin disorders such as benign lichenoid keratosis (BLK) [9] and seborrheic keratosis (SK) [10], and fibroadipose infiltrating lipomatosis [11]. Progressive segmental overgrowth in various regions of the body including visceral, subcutaneous, muscular, fibroadipose, and skeletal tissues is the common feature of these disorders [12]. The term, *PIK3CA*-related overgrowth spectrum (PROS), has been proposed to represent the broad range of clinical manifestations caused by *PIK3CA* mutations [13]. Similar and related disorders are caused by somatic mutations in the genes of the PI3K/AKT/mTOR (mammalian target of rapamycin) pathway, which include *PTEN* [14], *PIK3R2* (MCAP) [7], *AKT1* (Proteus syndrome) [15], *AKT2* (asymmetric overgrowth and hypoglycemia) [16], and *AKT3* (hemimegalencephaly) [17].

In this study, we identified the most common somatic mosaic mutation of *PIK3CA* (c.3140A>G, [p.H1047R]) in a Japanese female patient with PROS. We analyzed the correlation between the severity of overgrowth and the mutant allele frequency from affected tissues by quantitative PCR and direct sequencing. Further, we established dermal fibroblast cell lines harboring high and low frequencies of the mosaic mutation from the patient's affected tissues and assessed the therapeutic effects of four compounds (rapamycin, NVP-BEZ235, aspirin, and metformin) on PI3K/AKT/mTOR signaling inhibition by determining the phosphorylation status of associated proteins and measuring cell proliferation.

## RESULTS

### Patient

**Figure 1:**
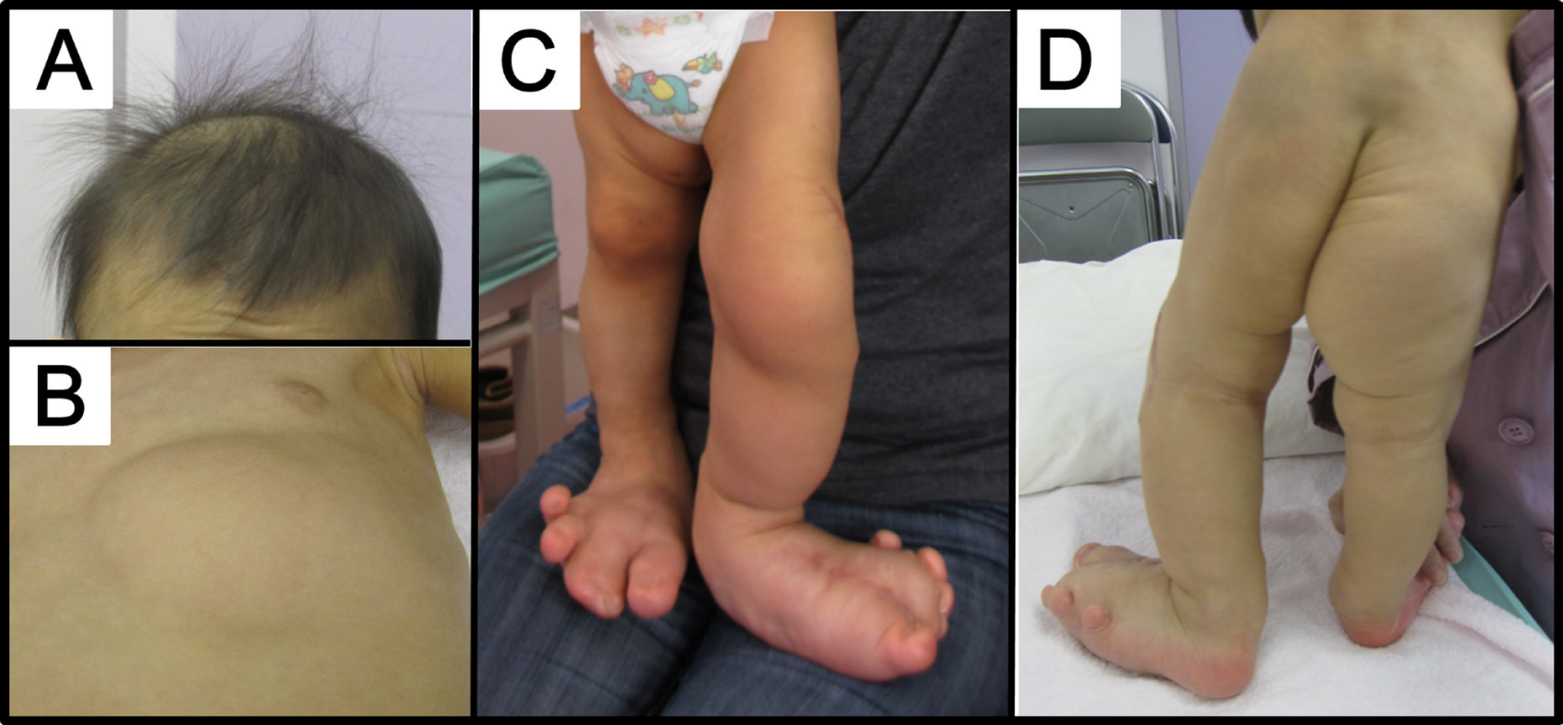
Patient (**A**) The patient had sparse hair at 2 years of age. (**B**–**D**) Note the asymmetric distribution of truncal lipomatous masses and bilateral lower extremity involvement. Lipomatous tissue was thin in other areas. Regional lipohypoplasia was seen in the lower extremities and chest.

The patient was a 3-year-old female born to non-consanguineous parents, a 34-year-old father and a 38-year-old mother (Figure 1). She was born at 39 weeks and 4 days gestation by normal vaginal delivery following an uneventful pregnancy. Her birth weight was 2988 g (−0.3 SD), and she measured 50.5 cm (0.4 SD) in height, with an occipitofrontal circumference (OFC) of 32 cm (−0.7 SD). Deformity of the feet was noted at birth. The left second, third, and fourth toes showed cutaneous syndactyly, and the right second and third toes showed syndactyly. An increased volume of subcutaneous adipose tissue in the left chest, the perineum, and the inside of the right thigh was obvious at one week after birth. The entire left leg and the right foot were severely enlarged, and the toes of both feet showed dactylomegaly (Figure 1C and Figure 1D). Brain magnetic resonance imaging at one month showed normal findings. The patient was diagnosed with bilateral dactylomegaly at 9 months and subcutaneous adipose tissue at the left knee and the distal phalanx of the first, second, and third left toes were surgically removed. At 2 years and 9 months, subcutaneous adipose tissue at the left knee, lower abdomen, perineum, and left lower leg was surgically removed. She showed normal milestones of head control at 3 months, sitting up at 5 months, and walking at 1 year. At 2 years, her weight, height, and OFC were 12.5 kg (0 SD), 85 to 89.5 cm (−1.4 SD∼0 SD, because of different sizes of the legs), and 46.5 cm (−1.1 SD), respectively. It was noted that she had sparse hair and that her skin was thin with a small volume of adipose tissue on parts of the body other than the regions of overgrowth. Intellectual development was normal as per age equivalency and development quotient at 2 years and 4 months was 111. The presented patient was diagnosed with PROS taking into account the following findings: 1) presence of somatic *PIK3CA* mutation (described below), 2) congenital onset, 3) overgrowth being sporadic and mosaic, 4) overgrowth in adipose and skeletal tissues, 5) isolated macrodactyly, overgrown splayed feet, and overgrown limb, and 6) truncal adipose overgrowth [13]. According to Martinez-Lopez et al. [18], this patient is categorized as CLOVES syndrome of PROS.

### Identification and characterization of the *PIK3CA* H1047R mutation

**Figure 2:**
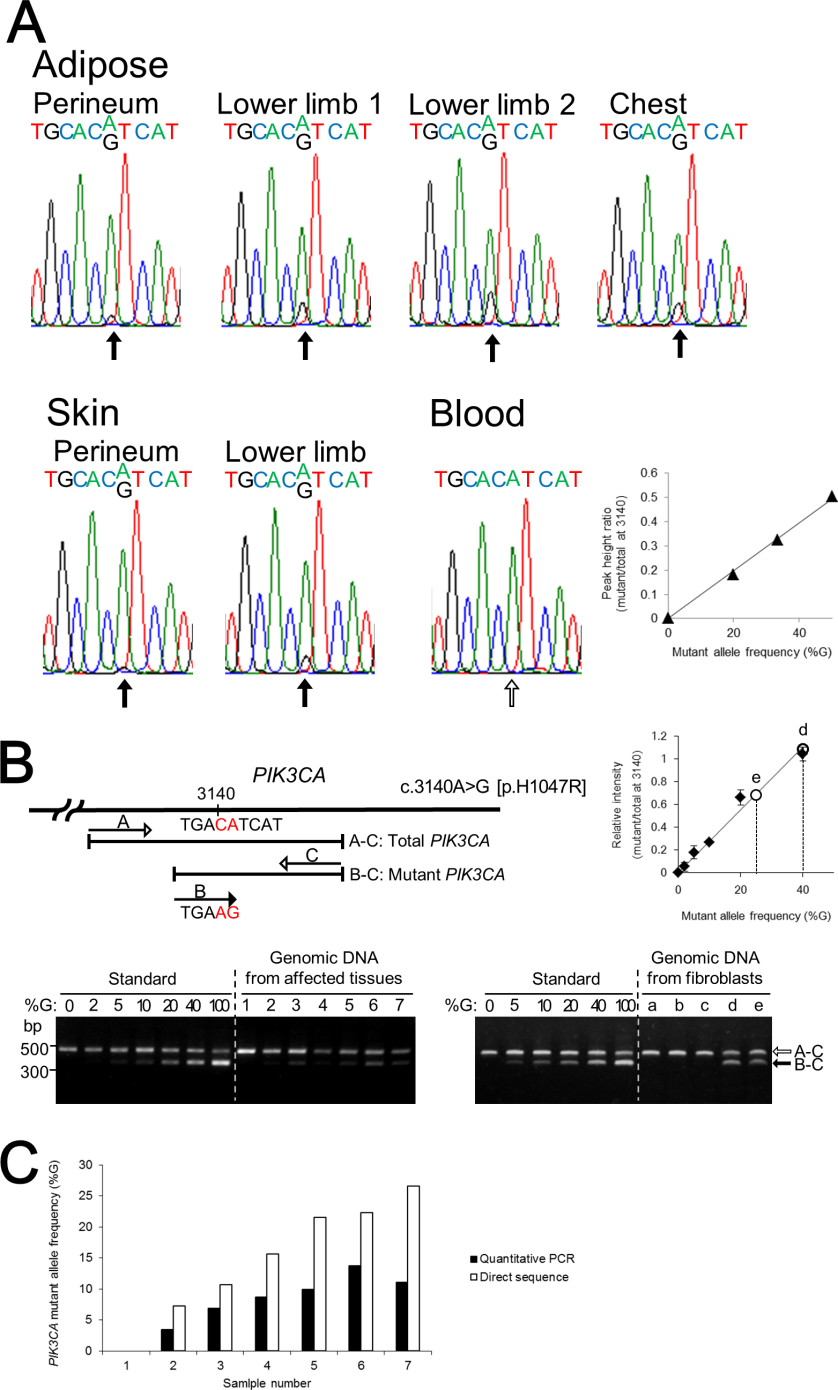
The identification and quantification of the mutation in *PIK3CA* (**A**) Direct nucleotide sequence analysis of *PIK3CA* was performed using isolated genomic DNA from blood and six different regions including subcutaneous adipose tissue and skin from the lower limbs. A mosaic mutation (c.3140A>G [p.H1047R]) in exon 20 was identified from affected adipose tissues and skin. The ratios of the mutant allele are different in the patient's affected tissues. Direct nucleotide sequence analysis was performed with plasmids containing normal or mutant *PIK3CA* fragment as standard reference materials. The right panel shows a standard curve for calculating *PIK3CA* mutant frequency by direct sequencing. (**B**) Multiplex PCR products of genomic DNA isolated from affected tissues, blood cells, and fibroblast cells were run through a 1.5% agarose gel and stained with ethidium bromide. Plasmids containing PCR products of wild-type and mutant *PIK3CA* exon 20 were used as standard reference materials. A 370-bp DNA fragment was generated from the mutant allele of *PIK3CA* (black arrow) and a 480-bp fragment was generated from both the wild-type and mutant *PIK3CA* alleles, as the internal control (white arrow). The sizes of the DNA markers are indicated on the left side. The upper right panel shows the standard curve for calculating *PIK3CA* mutant frequency by multiplex PCR. Lane: 1, blood; 2, skin from the perineum; 3, adipose tissue from the perineum; 4, skin from the lower limb; 5, adipose tissue from lower limb–1; 6, adipose tissue from the chest; 7, adipose tissue from lower limb–2; a, control fibroblast C2; b, control fibroblast C3; c, NHDF-c; d, PROS fibroblast from the skin of the lower limb; e, PROS fibroblast from the skin of the perineum. (**C**) *PIK3CA* mutant allele frequencies at nucleotide position 3140 in the patient's affected tissues and blood lymphocytes were calculated by quantitative multiplex PCR and direct sequencing. The x-axis labels are the same as in (B).

Since mutations in *PIK3CA* contribute to unilateral and/or segmental overgrowth phenotypes, we determined nucleotide sequences of *PIK3CA* from the patient's affected adipose tissues and skin by direct sequencing and identified a heterozygous mosaic mutation (c.3140A>G [p.H1047R]) in exon 20 (Figure 2A). The mutant allele was detected in all the affected tissues with the peak height ratio of A/(G+A) at nucleotide position 3140 determined by direct sequencing. Direct sequencing of PCR products and sequencing of 24 subcloned PCR products containing c.3140 confirmed that the patient's blood cells did not contain the mutant allele. To determine the mutant allele frequency at nucleotide position 3140 of *PIK3CA*, we performed a quantitative multiplex PCR assay (Figure 2B). The calculated mutant allele frequencies of *PIK3CA* in the patient's affected tissues and blood lymphocytes as determined by quantitative multiplex PCR and direct sequence are shown in Figure 2C. The frequencies of the *PIK3CA* mutant allele in the adipose tissue from the patient's perineum, lower limb 1, lower limb 2, and chest determined by quantitative multiplex PCR were 6.9%, 9.9%, 11.1%, and 13.7%, respectively, and those in the skin of the perineum and lower limb were 3.4% and 8.7%, respectively. Of note, mutant allele frequency ratios determined by direct nucleotide sequence analysis were approximately double the frequency ratios determined by quantitative PCR analysis. This is possibly because of the large cycle number (36) in the PCR protocol for amplifying the region of genomic DNA that included the mutation. The mutant allele frequencies were higher in subcutaneous adipose tissues than in skin tissue. Among the affected regions, the mutation frequencies were higher in the lower limbs and chest than in the perineum. Thus, there was a higher mutant allele frequency in samples from regions with a larger mass of subcutaneous adipose tissue.

### Effect of direct or indirect inhibition of PI3K/AKT/mTOR signaling in fibroblasts

**Figure 3:**
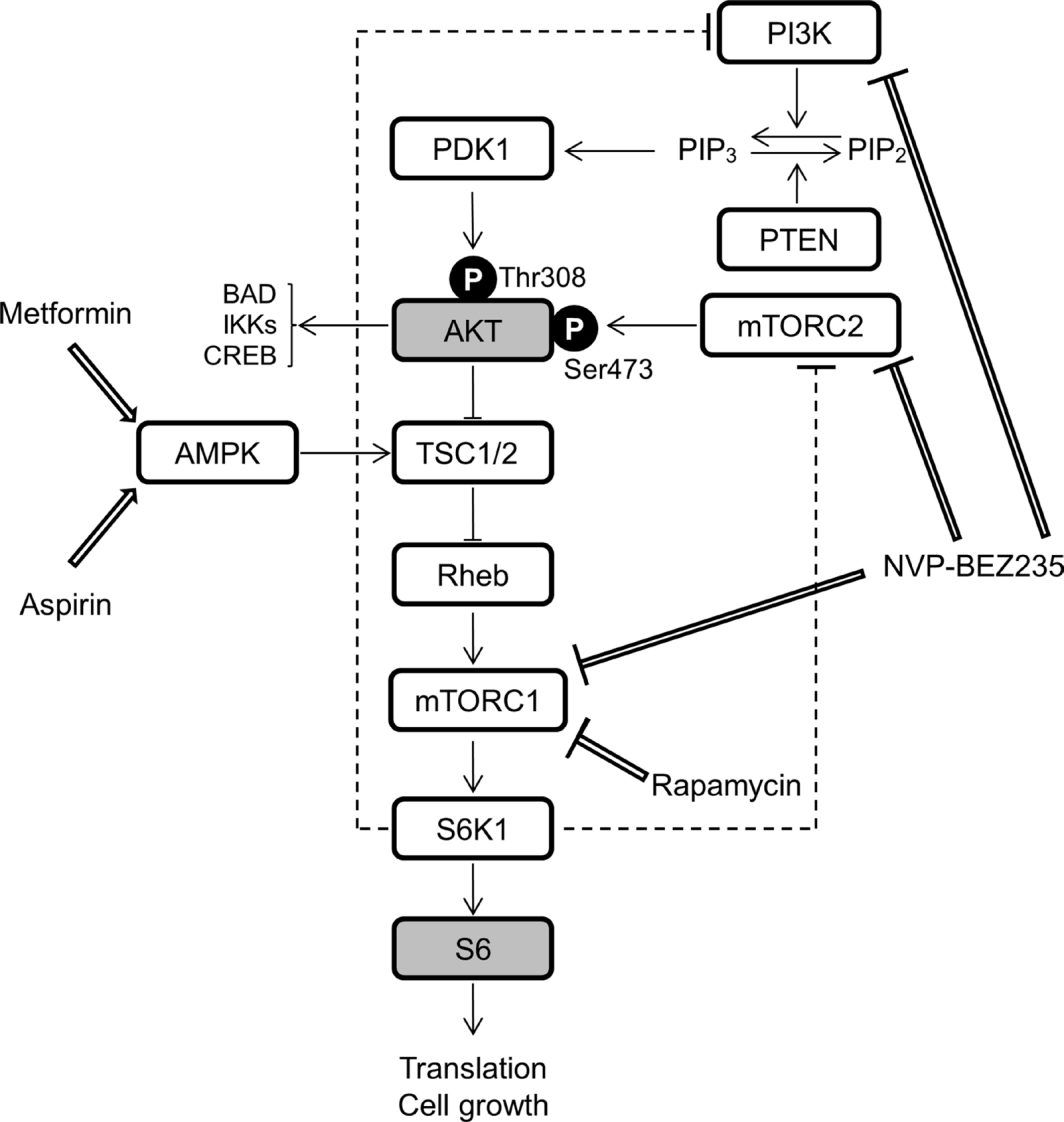
A schematic representation of simplified PI3K/AKT/mTOR signaling and the effects of rapamycin, NVP-BEZ235, aspirin, and metformin The following abbreviations are used: PTEN, phosphatase and tensin homolog; PDK1, 3-phosphoinositide dependent kinase 1; TSC1/2, tuberous sclerosis complex proteins 1 and 2; Pheb, Ras homolog enriched in brain; S6K1, ribosomal protein S6 kinase 1; BAD, BCL-2-associated death promoter; IKKs, IκB kinases; CREB, cyclic AMP response element binding protein.

**Figure 4:**
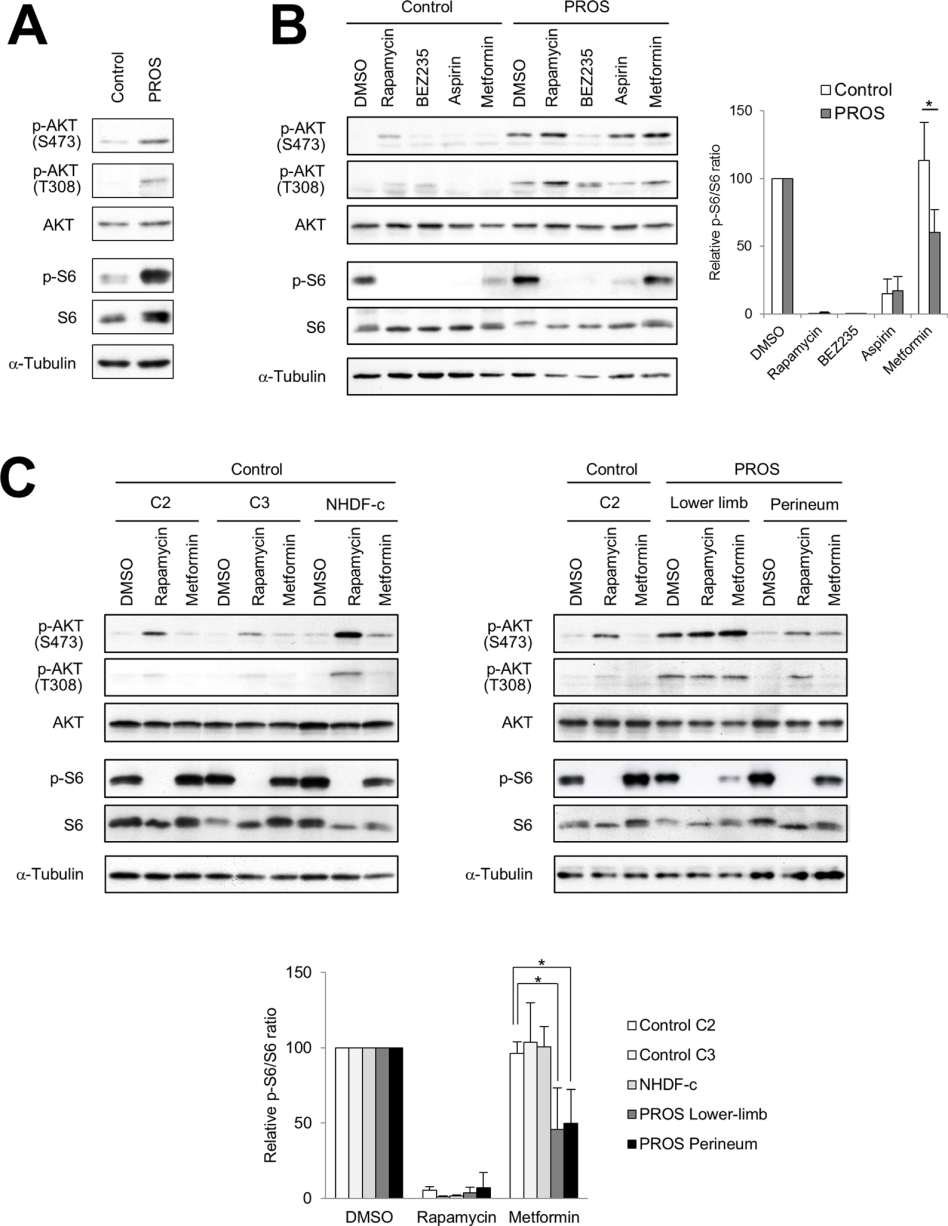
Effect of inhibitors of PI3K/AKT/mTOR signaling in fibroblasts (**A**) Immunoblot analysis of phospho-AKT (Ser473), phospho-AKT (Thr308), pan-AKT, phospho-S6 (Ser235/236), and pan-S6 in the PROS fibroblasts from the lower limb of the patient compared with control fibroblasts (C2). (**B**) Fibroblast cells were seeded 35-mm dished. After 24 hours, the control fibroblasts (C2) and PROS fibroblasts from the lower limb were treated with 1 nM rapamycin, 100 nM NVP-BEZ235, 5 mM aspirin, or 10 mM metformin for 2 days. (**C**) Control fibroblast lines C2 and C3, NHDF-c, and PROS fibroblast lines from the lower limb and perineum were treated with 1 nM rapamycin or 10 mM metformin for 2 days. In the right panel, extracts from control fibroblast line C2 were loaded as controls. Effects of these agents on the PI3K/AKT/mTOR pathway in fibroblast cells were analyzed by western blotting. α-Tubulin served as a loading control. Representative blots from three independent experiments are shown. All figures show the results of independent experiments. Quantification is presented as the relative ratio of phosphorylated S6 to total S6 in fibroblast cells. The quantification of the band intensity is normalized to the corresponding DMSO control treatment. Data are expressed as mean ± S.D. (*n* = 3). *p < 0.05, Student's *t-test*.

**Figure 5:**
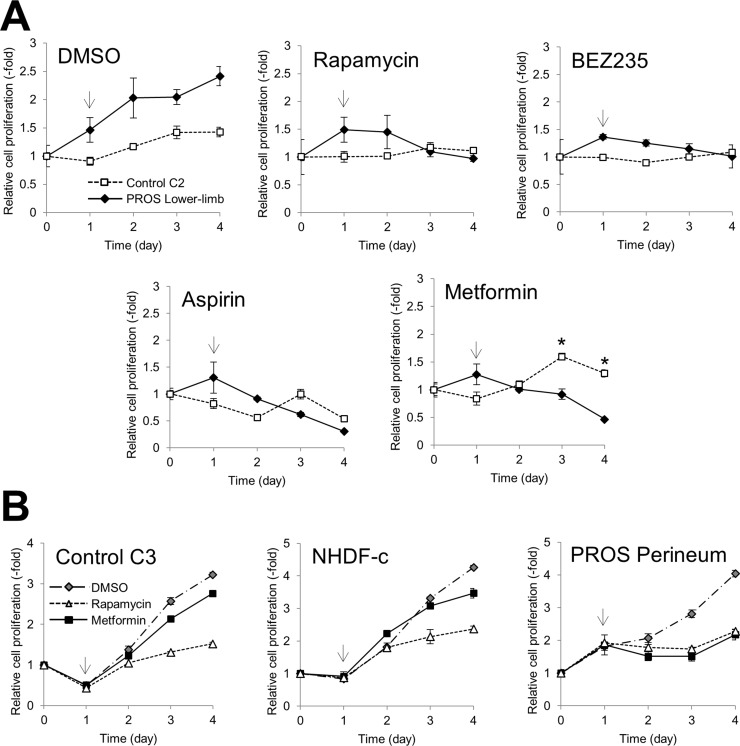
Effect of inhibitors of cell growth in fibroblasts Cell growth of PROS and control fibroblasts was assessed using the MTT assay. (**A**) Control fibroblasts C2 and PROS fibroblasts from the lower limb were treated with 1 nM rapamycin, 100 nM NVP-BEZ235, 5 mM aspirin, and 10 mM metformin. (**B**) Control fibroblast line C3, NHDF-c, and PROS fibroblasts from the perineum were treated with 1 nM rapamycin and 10 mM metformin. Arrows indicate the time point when the inhibitor was administrated. The data are normalized to Day 0. Data are expressed as mean ± S.D. (*n* = 3). *p < 0.05, Student's *t-test*.

Previous studies demonstrated that mutations in *PIK3CA* cause gain-of-function of PI3K, which leads to activation of downstream pathways and enhances cellular growth in cancer and a spectrum of overgrowth disorders [5, 19–21]. We hypothesized that inhibitors of the PI3K/AKT/mTOR signaling pathway could be used for effective therapeutic treatment for PROS. To evaluate the therapeutic effect of such compounds, we established dermal fibroblast cell lines from skins of the lower limb and perineum of the presented patient, as an experimental model. It is noted that, the cell lines from the lower limb and perineum have 40.9% and 25.8% mutant allele frequency ratios, respectively, as determined by quantitative multiplex PCR (Figure 2B). Immunoblot analysis revealed that the levels of phosphorylated AKT and ribosomal protein S6 (S6) were increased in PROS fibroblasts as compared to those in normal fibroblasts (Figure 4A). To assess the therapeutic effects on segmental overgrowth of PROS, we treated the two fibroblast lines derived from the patient's affected skin (PROS fibroblasts) and three normal controls (control fibroblasts) with rapamycin (mTOR inhibitor), NVP-BEZ235 (dual PI3K/mTOR inhibitor), aspirin, and metformin (Figure 3). We used metformin at the concentration used previously for cultured cancer cells [22–26]. First, we analyzed the changes in phosphorylation of the PI3K/AKT/mTOR signaling molecules S6 and AKT following treatment with these agents. Western blotting showed that rapamycin, NVP-BEZ235, and aspirin significantly inhibited phosphorylation of S6 in both PROS and control fibroblasts (Figure 4B and Figure 4C). Compare with rapamycin, metformin mildly reduced the level of phosphorylated S6 only in two of the PROS fibroblast lines (Figure 4C). In addition, NVP-BEZ235 decreased phosphorylation of AKT at Ser473 in PROS fibroblasts (Figure 4B). However, rapamycin induced phosphorylation of AKT at Ser473 in both control and PROS cells (Figure 4B and Figure 4C). Next, cell growth of PROS and control fibroblasts after treatment with each agent was assayed (Figure 5). Rapamycin, NVP-BEZ235, and aspirin showed remarkable growth inhibition of both PROS and control fibroblasts. Metformin also effectively suppressed the growth of both PROS fibroblast lines, which had different frequency of mosaic mutations. However, the effect on the growth of control cells was mild.

**Figure 6:**
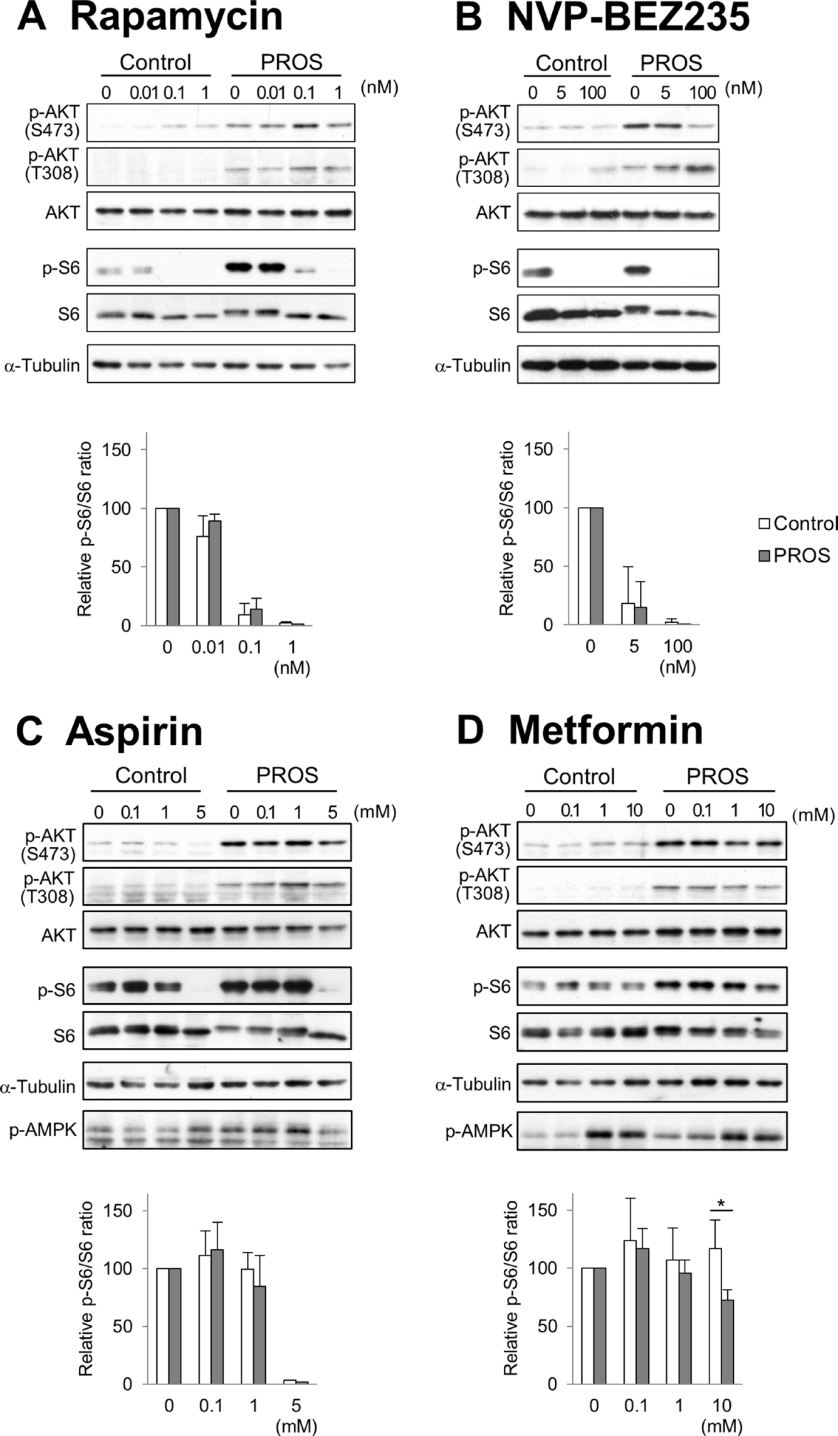
Dose-response study of the effects of inhibitors on PI3K/AKT/mTOR signaling After seeding the control fibroblasts (C2) and PROS fibroblasts from the lower limb, the cells were treated with different concentrations of rapamycin (**A**) NVP-BEZ235 (**B**) aspirin (**C**) and metformin (**D**) for 2 days. Activity of PI3K/AKT/mTOR was monitored by examining phosphorylation of AKT-S473, AKT-T308, and S6 by western blotting. AMPK activation was monitored by phosphorylation of threonine 172 with a phosphor-specific antibody. α-Tubulin served as a loading control. Representative blots from three independent experiments are shown. Quantification is presented as the relative ratio of phosphorylated S6 to total S6 in the control (white boxes) and the PROS (gray boxes) fibroblast cells. The quantification of the band intensity is normalized to the corresponding DMSO control treatment. Data are expressed as mean ± S.D. (*n* = 3). *p < 0.05, Student's *t*-tests.

To examine the effect of these compounds on PI3K/AKT/mTOR signaling in detail, dose-response studies were carried out on control (C2) and PROS (lower limb) fibroblasts (Figure 6). Western blots from fibroblasts treated with rapamycin show that there was an inverse relationship between the phosphorylation of S6 and AKT. NVP-BEZ235 diminished phosphorylated S6 and AKT at Ser473 in PROS fibroblasts, but inhibition of AKT phosphorylation at Ser473 required higher concentrations of NVP-BEZ235 than that of S6 phosphorylation. Although NVP-BEZ235 also inhibited PI3K activity, phosphorylation of AKT at Thr308, which is phosphorylated by PDK1, was induced by treatment of 100 nM NVP-BEZ235 in PROS fibroblasts. High concentrations of aspirin and metformin (5 mM and 10 mM, respectively) were required for inhibition of S6 phosphorylation on PROS cells. Since it is known that metformin and, in some cases, aspirin can activate AMP-activated protein kinase (AMPK) [27, 28], the phosphorylation state of AMPK in the fibroblasts was investigated. Metformin at concentrations of 1 mM and higher induced phosphorylation of AMPK of PROS and control fibroblasts, while aspirin did not affect AMPK phosphorylation in PROS and control fibroblasts at the concentrations tested.

## DISCUSSION

We report the characterization of a PROS patient harboring a mosaic *PIK3CA* (c.3140A>G [p.H1047R]) mutation in the affected tissues. H1047R is the most common amino acid substitution, occurring in a helix domain at the end of the activation loop of the p110α/p85α complex. A recent study demonstrated that the H1047R mutation in PIK3CA causes extensive remodeling of gene signatures, thus this single base mutation has an unexpectedly deep and broad impact on the phenotypic properties of the cell [29]. Therefore, it is important to investigate whether the patient's tissues contain this mutation. We demonstrate the correlation between mutant allele frequency and the size of adipose tissues in the presented patient. Moreover, we also show that direct sequencing is a useful diagnostic genetic test to identify a mosaic mutation in affected adipose tissue or fibroblast cells, even though these are small overgrown regions.

A major manifestation of PROS is progressive segmental overgrowth in various regions of the body present congenitally or starting in early childhood [13]. Since surgical debulking and orthopedic procedures are currently the only available treatments for patients with segmental overgrowth syndromes [30, 31], it is imperative to develop new therapeutic approaches and effective treatments to combat these syndromes. Recently, the therapeutic effects of small compounds on the inhibition of the PI3K/AKT/mTOR signaling pathway for venous malformation, lymphatic malformations (LMs), and PROS with *PIK3CA* mutants have been reported [32–34]. These studies have reported the following findings: 1) Treatment with rapamycin and BYL719 (a PI3K p110α specific inhibitor) diminished AKT phosphorylation in human umbilical vein endothelial cells (HUVEC) expressing *PIK3CA* mutants cultured in a medium containing 10% fetal calf serum (FCS). However, the effect on cell proliferation was not described. 2) Rapamycin and GDC-0941 (a potent inhibitor of PI3K p110α) effectively inhibited the proliferation of both LM-mutant and control lymphatic endothelial cells (LECs). Rapamycin had a stronger effect on proliferation of LM-LECs than that of control LEC, but the effect of rapamycin and GDC-0941 on the phosphorylation of AKT in LM-LECs was not described. 3) Wortmannin and LY294002 (PI3K inhibitors) abrogated the over-activation of AKT and p70S6K in fibroblasts derived from two PROS patients in the absence of serum. Thus, wortmannin and LY294002 decrease growth factor-independent proliferation and AKT phosphorylation. However, the effect of these inhibitors on the growth of normal cells was not described.

Constitutive activation of PI3K is a characteristic feature in PROS patients with *PIK3CA* mutations and overgrowth of cells in patients’ organs usually occurs with the onset of expression of certain growth factors during development. Previous studies have shown PI3K activation of lymphoblastoid cells harboring *PIK3CA* mutations cultured in media with 15% FCS [7, 35]. Therefore, we established PROS patient-derived fibroblasts harboring a high frequency (40.9%) and a low frequency (25.8%) of the mutation to study the therapeutic effects of small compounds on control and PROS fibroblasts in medium containing 10% FCS. First, we analyzed the effects of the direct inhibitors of PI3K/AKT/mTOR signaling pathway, rapamycin and NVP-BEZ235, on PROS fibroblasts. Rapamycin acts as a specific inhibitor of mTORC1 (Figure 3) and has synergistic anti-cancer effects [36]. NVP-BEZ235 is a dual PI3K and mTOR inhibitor [37] (Figure 3) and is currently in phase I/II clinical trials for treating advanced solid tumors [38]. In this study, we show that 1 nM rapamycin completely suppressed the phosphorylation of S6 and the cell growth of both control and PROS fibroblasts (Figures 4, 5, and 6). Thus, the PROS fibroblasts are sensitive to a low concentration of rapamycin, which is a similar dose as is used to treat rapamycin-sensitive cancer cells [39]. In this model, rapamycin increased phosphorylated AKT, likely as a result of rapamycin-induced feedback activation of AKT signaling [40] (Figures 3, 4C, and 6A). Thus, PI3K/AKT signaling upstream of mTOR is more highly activated in PROS fibroblasts than in control cells, leading to the activation of the collateral pathways upstream of mTOR. Similarly, 5 nM NVP-BEZ235 inhibited mTOR activity of control and PROS fibroblasts by completely suppressing phosphorylation of S6 while slightly increasing the phosphorylation of AKT at Thr308 in PROS fibroblasts (Figures 3, 4B, and 6B). This phenomenon could be explained by induction of feedback activation of AKT signaling by mTOR inhibition. Importantly, the findings of this study are similar to those of a previously reported study on cancer cells with a PI3K active mutation [41]. Thus, rapamycin and NVP-BEZ235 are therapeutically effective to treat PROS, but these inhibitors suppress cell growth of both PROS cells and control cells. Moreover, side effects such as mucositis, elevation of ALT/AST, hypercholesterolemia, headache, and neutropenia were reported in the treatment of complicated vascular anomalies in children with rapamycin [42]. These findings indicate that administration of both compounds should be carefully performed to avoid serious side effects in growing children.

Next, we analyzed the effect of indirect inhibitors of the PI3K/AKT/mTOR signaling pathway aspirin and metformin on control and PROS fibroblasts. Aspirin is an anti-inflammatory drug and has been shown to increase the five-year survival rate of patients with several types of cancer [43], including colorectal cancer harboring *PIK3CA* mutations in exons 9 and 20 [44, 45]. Aspirin inhibits cyclooxygenase (COX), leading to reduction of prostaglandin production [46]. Recent studies have shown that aspirin inhibits mTOR signaling by activating AMPK in cancer cells [28, 47] (Figure 3). We demonstrated that 5 mM aspirin completely blocked the phosphorylation of S6 and inhibited cell growth of control and PROS fibroblasts, but we did not observe phosphorylation of AMPK in PROS cells (Figures 4B, 5A, and 6C). Importantly, the aspirin concentration (5 mM) used in this study is similar to the effective aspirin concentration (1 to 3 mM) in the blood of patients taking high-dose aspirin (100 mg/kg/day) for type 2 diabetes and Kawasaki disease [48, 49]. However, the use of high-dose aspirin may increase the risk of gastrointestinal hemorrhage [50], sensorineural hearing loss [51], and Reye syndrome [52] in children. Metformin (1,1-dimethylbiguanide hydrochloride), a biguanide derivate, is the drug most commonly used in treating type 2 diabetes [53]. Previous epidemiological studies have demonstrated that metformin can reduce the risk of cancer in diabetic patients [54, 55]. Recently, metformin has been shown to decrease the risk of several cancers, including colon, breast, prostate, and pancreatic cancers [56]. One of the molecular mechanisms underlying the anti-cancer effects of metformin is the activation of AMPK (Figure 3), which results in suppression of mTOR signaling [56]. In this study, we found that 10 mM metformin treatment activates AMPK and reduces S6 phosphorylation without affecting AKT phosphorylation in both control and PROS fibroblast cells (Figure 6D). Importantly, metformin strongly inhibited cell growth of the PROS fibroblasts containing either high or low frequency of the mosaic H1047R mutation, while the effect on the three control cell lines was mild (Figure 5). Compared with the other three compounds, metformin had a milder effect of suppressing phosphorylation of S6 in PROS cells (Figure 4 and Figure 6). Therefore, the suppression of the phosphorylation of S6 is difficult to explain in light of the selective cell growth inhibition of PROS cells by metformin, and so, other molecular mechanisms may be involved in the selective growth inhibition. A high concentration of metformin (∼10 mM) was necessary to suppress the growth of PROS fibroblasts in this study likely because of the fact that metformin does not passively diffuse through cell membranes because of its low lipophilicity and so it must be transported into cells by the transmembrane protein organic cation transporters 1-3 [57–59]. Similarly, a concentration of at least 1 mM metformin was required to suppress the growth of two types of endometrial cancer-derived cell lines, one of which has a mutation in PTEN in the PI3K/AKT/mTOR signaling pathway [60] (Figure 3). On the other hand, a recent clinical study on patients with endometrial cancer has also shown that oral administration of 1500–2250 mg/day of metformin for 4 weeks resulted in a significantly reduced expression of the cell proliferation marker Ki-67 and decreased phosphorylated S6 in endometrial cancer tissues [60]. The metformin concentrations in the plasma and endometrial cancer tissues of these patients were 6.8 to 18.1 μM and 1.2 to 5.1 μmol/kg wet weight, respectively [60]. Although we do not have sufficient evidence to explain the difference in effective metformin concentrations, low concentrations having been reported in patients’ blood and tissue compared with high concentrations required for efficacy in cell culture medium for PROS fibroblasts in this study, it is possible that metformin acts on PROS fibroblasts with different frequencies of mosaic mutations in the same manner as it does in cancer cells [60]. In a randomized clinical trial of metformin to treat obese insulin-resistant children with dosages ranging from 500 to 2000 mg/day, side effects such as nausea, loose stool, and fatigue were reported, but serious or life-threatening events were not noted [61]. Therefore, administration of regular daily doses of metformin is one of the candidate treatments for PROS patients.

In summary, we studied the effect of four small compounds, rapamycin, NVP-BEZ235, aspirin, and metformin on control and PROS fibroblasts and demonstrated that all four compounds could suppress the activated PIK3/AKT/mTOR signaling pathway and the growth of PROS patient-derived fibroblasts by suppressing phosphorylation of S6. However, only metformin showed mild inhibition of the growth of control fibroblasts. Since PROS is a developmental disorder in children, metformin is a candidate drug for treating PROS. However, further investigation is necessary to elucidate the molecular mechanism governing the higher sensitivity of cell growth of PROS fibroblasts to metformin than that of control cells and to determine the optimal concentration of metformin required to effectively suppress the growth of mutant cells in PROS patients.

## MATERIALS AND METHODS

### Bioethics approval

Written informed consent was obtained from the family members who participated in this study. The study was conducted after approval from the institutional review board at the Institute for Developmental Research, Aichi Human Service Center and in accordance with the principles embodied in the Declaration of Helsinki.

### Identification of somatic mutation

Genomic DNA was isolated from seven different regions, including subcutaneous adipose tissue from the chest, lower limb, and perineum, skin from the lower limb and perineum, and white blood cells by phenol/chloroform extraction. To identify the mutations in *PIK3CA*, DNA fragments including all exons and exon-intron junctions of *PIK3CA* were amplified from genomic DNA by 36 cycles of polymerase chain reaction (PCR) using specific primer sets. The resulting PCR products were separated by agarose gel electrophoresis, purified with QIAEX II Gel Extraction Kit (Qiagen, Hilden, Germany), and then directly sequenced using the GenomeLab GeXP Genetic Analysis System (AB SCIEX, Framingham, MA, USA) and the GenomeLab Dye Terminator Cycle Sequencing with Quick Start Kit (Beckman Coulter, Brea, CA, USA) with 50 to 100 fmol of template DNA. For quantification, 380 bp of wild-type and mutant PCR products of the *PIK3CA* gene including exon 20 were amplified from genomic DNA isolated from lower limb adipose tissue by PCR using a specific primer set (sense S1: 5′-caatgatgcttggctctgga-3′; antisense A1: 5′-tcaaaccctgtttgcgtttac-3′) and subcloned into pGEM-T Easy vector (Promega, Madison, WI, USA).

### Determination of H1047R frequency in tissues

To determine frequencies of the missense mutation of *PIK3CA* gene (c.3140A>G) in patient tissues, we established a quantification method for determining the dosage of genomic DNA using multiplex PCR (Figure 2B). Specific primer pairs (mismatch sense S2: 5′-atgaaacaa atgaatgatgcaag-3′ and antisense A2: 5′-acaaacaatcttcaaagt ttacct-3′; --cacg was changed to --caag) were designed to specifically amplify a 370 bp DNA fragment containing only the mutant *PIK3CA* gene (c.3140A>G). Another pair of primers (S1 and A2) were also designed to amplify a 480 bp genomic DNA fragment containing wild-type and mutant *PIK3CA*. Aliquots (0.05 μg) of genomic DNA from each affected tissue and a normal blood sample were amplified by PCR in a total volume of 20 μL, containing 0.45 μM of total primers, 30 mM of each dNTP, 2.5 mM MgCl_2_, 10 mM Tris–HCl (pH 8.3), 50 mM KCl, and 1 U of AmpliTaq-Gold (Applied Biosystems). The ratio of the concentrations of the three primers was 1 : 4 : 5 (S1 : S2 : A2). PCR samples were preheated at 94°C for 10 min and 32 PCR cycles were performed. Each cycle consisted of 30 s denaturation at 94°C, 30 s annealing at 56°C, and 30 s extension at 72°C. The PCR products were electrophoresed on 1.5% agarose gels and band intensities were measured using Image Quant TL 7.0 software (GE healthcare).

### Treatment of PROS fibroblast cells with inhibitors of the PI3K/AKT/mTOR signaling pathway

Two PROS fibroblast cell lines were established from the patient's lower limb and perineum. Two control fibroblast cell lines (C2 and C3), which were previously established from the skins of two unrelated healthy volunteers of a 36-year-old male and a 27-year-old female, respectively, [62] were used. Additionally, a normal human dermal fibroblast cell line established from the skin of a 14-year-old male (NHDF-c, c-12300) was purchased from PromoCell GmbH (Heidelberg, Germany). Fibroblast cells were cultured as previously described [62]. Briefly, fibroblasts were maintained in Eagle's minimal essential medium (EMEM; Sigma-Aldrich, St. Louis, MO, USA) supplemented with 10% FCS, penicillin (100 U/mL), streptomycin (0.1 mg/mL), L-glutamine (1.75 mM), HEPES (10 mM), and NaHCO_3_ (0.13%). All supplemental reagents, except for FCS, were purchased from Nacalai Tesque (Kyoto, Japan). The cells were plated on 35-mm collagen-coated dishes at a density of 1.1 × 10^4^ cells/cm^2^ for biochemical experiments. Fibroblast cells were treated with rapamycin (LKT Laboratories, St. Paul, MN, USA), NVP-BEZ235 (Chemscene, Monmouth Junction, NJ, USA), aspirin (acetylsalicylic acid; Wako, Osaka, Japan) and metformin hydrochloride (Sigma-Aldrich) for 2 days, washed with PBS, and then harvested and subjected to SDS-PAGE and western blotting.

### Western blotting

Samples were electrophoresed on 11% SDS polyacrylamide gels and transferred to polyvinylidene difluoride membranes (Merck Millipore, Darmstadt, Germany). The membranes were incubated in 5% bovine serum albumin in Tris-buffered saline containing Tween-20 (50 mM Tris-HCl pH 7.4, 150 mM NaCl, and 0.03% Tween-20) and then incubated with primary antibodies anti-pan-AKT, anti-phospho-AKT (Ser473), anti-phospho-AKT (Thr308), anti-pan-S6, anti-phospho-S6 (Ser235/236) (Cell Signaling Technologies, Danvers, MA, USA), and anti-α-tubulin (Sigma-Aldrich), followed by incubation with a horseradish peroxidase-conjugated secondary antibody. Immunoreactive proteins were visualized with Western Lightning Plus-ECL (PerkinElmer, MA, USA). Densitometric measurement of bands on western blotting was performed using ImageJ software (US National Institutes of Health, Bethesda, MD, USA).

### MTT assay

Cell growth was assessed using the 3-(4,5-dimethylthiazol-2-yl)-2,5-diphenyltetrazolium bromide (MTT) assay. Briefly, fibroblast cells were seeded at a density of 3.1 × 10^4^ cells/cm^2^ onto collagen-coated 96-well culture plates. Each day, 10 μL of MTT solution (5 mg/mL in PBS) was added to each well. After incubation for 2 h at 37°C, the purple-blue MTT formazan precipitate was dissolved in 200 μL of MTT solvent (40 mM HCl in isopropanol) and the absorbance was measured with a multiplate reader at 570 nm.

### Statistical analysis

Data are expressed as means ± SD. Statistical analysis was performed by Student's *t-test*. A *p value* of < 0.05 was considered to be statistically significant.

## Abbreviations

AMPK: AMP-activated protein kinase
COX: cyclooxygenase
mTOR: mammalian target of rapamycin
MTT: 3-(4,5-dimethylthiazol-2-yl)-2,5-diphenyltetrazolium bromide
PI3K: phosphatidylinositol 3-kinase
PIK3CA: phosphatidylinositol-4,5-bisphosphate 3-kinase catalytic subunit alpha
PROS: *PIK3CA*-related overgrowth spectrum
S6: ribosomal protein S6.
